# Metabolic Swifts Govern Normal and Malignant B Cell Lymphopoiesis

**DOI:** 10.3390/ijms22158269

**Published:** 2021-07-31

**Authors:** Aikaterini Poulaki, Stavroula Giannouli

**Affiliations:** 1 Department of Pathophysiology, School of Medicine, National and Kapodistrian University of Athens, 11527 Athens, Greece; aikaterini.poulaki@gmail.com; 2 Hematology Unit, Second Department of Internal Medicine, School of Medicine, National and Kapodistrian University of Athens, 11527 Athens, Greece

**Keywords:** metabolism, lymphopoiesis, B cell, adaptive immunity, mitochondria, Warburg effect, glycolysis, lymphomagenesis, lymphoma

## Abstract

B lymphocytes are an indispensable part of the human immune system. They are the effective mediators of adaptive immunity and memory. To accomplish specificity against an antigen, and to establish the related immunologic memory, B cells differentiate through a complicated and strenuous training program that is characterized by multiple drastic genomic modifications. In order to avoid malignant transformation, these events are tightly regulated by multiple checkpoints, the vast majority of them involving bioenergetic alterations. Despite this stringent control program, B cell malignancies are amongst the top ten most common worldwide. In an effort to better understand malignant pathobiology, in this review, we summarize the metabolic swifts that govern normal B cell lymphopoiesis. We also review the existent knowledge regarding malignant metabolism as a means to unravel new research goals and/or therapeutic targets.

## 1. Introduction

B lymphocytes are an indispensable part of the mammalian immune system. They effectively mediate maybe its most impressive function, the adaptive immune response and memory [1,2]. B cell physiology has been and to a great extent still remains, despite vivid research, elusive. This fact largely reflects upon the complicated and elaborate process of the specific for any antigen (Ag), antibody (Ab) assembly and secretion. Humoral immunity involves both stochastic and tightly regulated events initiated inside the bone marrow (BM), that are eventually completed within the secondary lymphoid organs, lymph nodes, spleen and the widespread mucosa-associated lymphoid tissue. The active training of B cells to recognize specific Ag, to keep a memory of their encounters and to maintain the ability to synthesize and secrete specific Abs indefinitely for an infinite number of different molecules encompasses risky drastic genetic modifications. Double stranded breaks (DSB) are indispensable in the B cell lifestyle [3,4]. They are induced in a random manner at multiple time points during the B cell development in order for the assembly of an avid, non-self-reactive B cell receptor (BCR), or Ab [5]. The fact that normal B cells poise repeatedly between genomic stability and induced instability to accomplish their role is unique to this lineage. Successful genetic modifications are positively selected and followed by periods of intensive cellular expansion, rendering B cells the most rapidly proliferating cells inside the healthy human [6].

**Figure 1:**
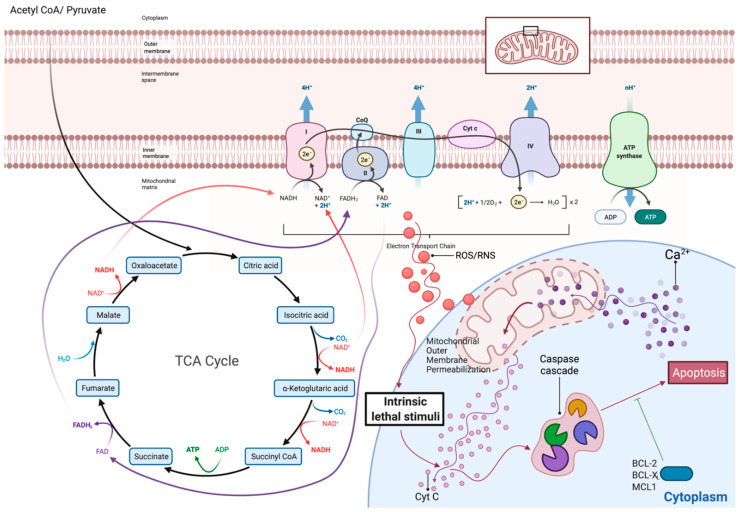
Overview of mitochondria tricarboxylic acid cycle (TCA) and electron transport chain (ETC). Coupling of TCA with the ETC is named oxidative phosphorylation (OxPhos) and is the main metabolic source of intracellular energy in the form of ATP. During the ETC, pooling of electrons (e^−^) inside the ETC proteinic complexes, for instance due to imbalances between electron feeding and ETC capacity, leads to release of reactive oxygen species (ROS). To protect the cell from the effects of this oxidative stress, mitochondrial existence is coupled with an intrinsic ability of the organelle to induce cellular death, when its function is compromised. Ca^+2^ and/or other stimuli activate this intrinsic apoptotic pathway, which leads to increased mitochondrial outer membrane permeabilization. Release to the cytoplasm of the normal mitochondrial membrane-bound cytochrome c (Cyt c) activates a positive caspase regulatory cascade that leads to apoptosis. Pro-survival proteins belonging to the BCL-2 family (BCL-X, MCL-1, BCL-2) rescue cells from such events and are a major factor in both normal and malignant B cells’ physiology. Detailed analysis of the mechanisms of action of such antiapoptotic proteins is beyond the scope of this review and will therefore not be discussed further. Adapted from “Electron Transport Chain”, by BioRender.com (2020). Retrieved from https://app.biorender.com/biorender-templates (accessed on 6 July 2021).

In order to maintain normality and avoid malignant transformation, the B cell training, differentiation and secreting program is stringently controlled through numerous cell intrinsic checkpoints as well as microenvironmental influence [7,8]. One of the most impressive and surprisingly underappreciated such control programs is mediated through massive bioenergetic swifts [8]. Changes in metabolism are either the result or the driver of induced replication or quiescence during the B cell development. Failure of the differentiating precursor to establish metabolic adequacy usually in the form of mitochondrial integrity activates a deadly cascade that results in mitochondrially induced apoptotic death (Figure 1) [9]. Metabolic swifts render the B cell precursors susceptible or in need of further signaling in order to proceed to their next stage of differentiation, or otherwise perish [10].

**Figure 2:**
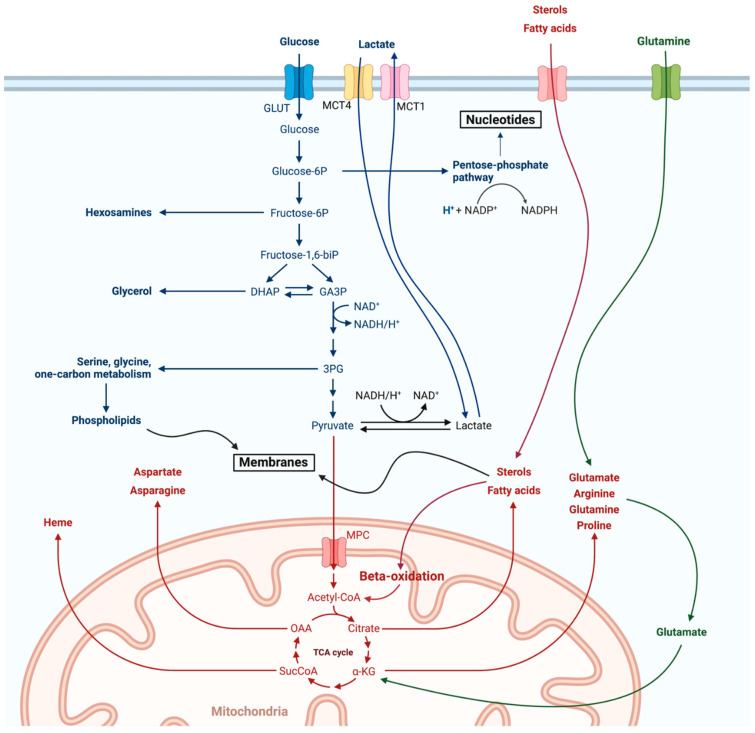
Overview of cellular metabolism. Both the Warburg glycolysis (blue) as well as mitochondrial OxPhos (red) are shown here along with their major branching and/or feeding pathways. Anaplerosis of the TCA through glutaminolysis is of special interest as it will be discussed further in the article and is depicted in green. Adapted from “Warburg Effect”, by BioRender.com (2020). Retrieved from https://app.biorender.com/biorender-templates (accessed on 6 July 2021).

B lineage, as every cell’s bioenergetic program, involves two major pathways that are in a dynamic balance with one another according to the stage of cellular differentiation and the microenvironmental signaling: aerobic glycolysis at one end and mitochondrial oxidative phosphorylation (OxPhos) at the other [11]. Aerobic glycolysis, or the Warburg effect, summarizes the extramitochondrial utilization of glucose for inefficient energy production in the form of ATP, though with the major benefit of redox state improvement, NADPH production and active anabolism (nucleotides through the pentose phosphate pathway (PPP), phospholipids, etc.). Mitochondrial OxPhos on the other hand involves the sequential oxidation of carbonic molecules, glucose in the form of pyruvate, fatty acids, amino acids, etc., through the mitochondrial tricarboxylic acid cycle (TCA) [12]. Electrons that are released inside TCA are carried by coenzyme molecules, namely FADH2 and NADH, towards a mitochondrial membrane-bound four-proteinic complex sequence, the electron transport chain (ETC) [13]. The ETC couples with redox reactions to allow electron flow through its complexes, creating an electrochemical gradient that is eventually released from a fifth proteinic complex, the ATP synthase (or Complex V), through ATP synthesis and H_2_O release. Coupling of the TCA with the ETC, in other words the OxPhos, is the most efficient way of producing energy. As with all biological systems, OxPhos is not perfect. Oxygen is used as the electron carrier through the ETC. Due to inherent flaws in the ETC, electrons periodically pool in complexes. This results in the formation and release of reactive oxygen species (ROS) [14]. ROS are by definition nucleophilic intermediates, and therefore actively attack several intracellular molecules, including nucleic acids. In small amounts, though, they play a pivotal signaling function, communicating the conditions in the cellular energy factory to the nucleus [13,15]. Of note, release of the dangerous ROS is avoided during the Warburg effect (Figure 1 and Figure 2).

The balance between these two major pathways is extensively and uniquely manipulated by the B cell lineage during differentiation [16]. In this article, we will therefore review the complicated process of B cell to antibody-secreting cell (ASC or plasma cell (PC)) or memory B cell (MBC) differentiation, underlining the bioenergetic aspect of the process. As B cell lymphomas, the major B cell-related malignancies, rank 8th in the 2020 WHO Globocan (global cancer observatory) fact sheet, we will review the metabolic changes that characterize them [17]. With rare exceptions, B cell lymphomas represent immortalized “frozen” B cell developmental stages, with tumorous populations retaining many of their normal counterparts’ features, the vast majority of them being related one way or another to recombination events in the process of BCR formation [18,19,20]. Understanding the normal bioenergetic physiology of the B cell will, as it is already increasingly evident, shed light on the mysterious life of this lineage [19,21]. Furthermore, comparison with their malignant counterparts, as it will be attempted in this review, is crucial for unraveling lymphomatous pathophysiology and thus new therapeutic targets and strategies [22].

## 2. Physiologic B Lymphopoiesis

### 2.1. From the HSC to the Small Pre-B Cell: Engagement in the B Cell Fate

As with all hematopoietic cells, the precursor of the B cell lineage is the hematopoietic stem cell (HSC). Either short-lived (SL-HSC) or long-lived HSC (LL-HSC) divide symmetrically (or asymmetrically only for the LL-HSC) to give multipotent precursors (MPP) that are not yet lineage-committed but cannot self-renew [23,24]. Up to this point, little is known about the metabolism of these precursors. To maintain their quiescence and to ensure long-term health, stem cells are reliant on Warburg metabolism. Indeed, their minimal ATP requirements and their residency in hypoxic BM niches facilitate this lifestyle. Despite the common misconception, stem cells do harbor a confined mitochondrial cargo. The hypoxic microenvironment prevents OxPhos and thus ROS release. Moreover, their niches ensure that stem cells maintain maximal health within their energy factories by providing them with new healthy organelles [25].

**Figure 3:**
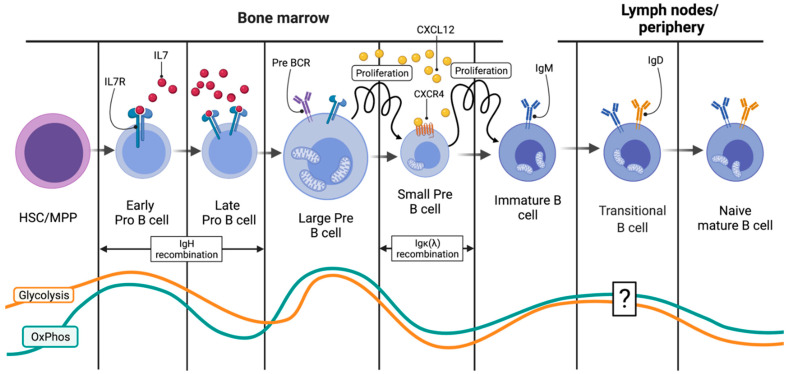
The first half of B cell lymphopoiesis happens inside the BM and results in immature IgM-expressing B cells in the periphery. Visual summary of the initial events that lead to the formation of IgM^+^ immature B cells and that happen inside the BM. Close control of differentiation and replication is needed to avoid malignant transformation during active Ig recombination. This is achieved through both tight metabolic regulation as well as sequential trafficking of the B cell lineage between specific BM niches (from the IL7-rich to the CXCL12 one). Bioenergetic swifts are of particular interest and are specifically shown in this diagram as orange (glycolysis) and blue (OxPhos) lines. IL7(R): Interleukin 7 (receptor), CXCR4: C-X-C Motif Chemokine Receptor 4, CXCL12: C-X-C Motif Chemokine Ligand 12. Created with BioRender.com.

Specialized niches inside the BM microenvironment engage the MPP through sequential trafficking between them, cell–cell interactions and cytokine release to a B cell fate. The details of this complicated process are still under investigation. For the lymphoid lineage at least, SDF-1 (Stromal-Derived Factor 1 or CXCL12) and its receptor on the differentiating progenitor CXCR4 are considered indispensable of its homing [23]. A portion of the VCAM^neg^ (vascular cell adhesion protein 1) MPP precursors through yet unknown signaling pathways start expressing increasing levels of IL7R while decreasing CXCL12. Thus, they move towards neighboring IL7-rich reticular cell niches [6]. The efficient IL7R–IL7 interaction marks lymphoid lineage, and more precisely, the T/B cell fate commitment as it initiates a STAT_5_ (Signal Transducer and Activator of Transcription 5)-based cascade that results in the expression of RAG1/2 (recombination-activating genes) complex in a fraction of the common lymphoid progenitors (CLP) [6,26]. Thereafter, transcriptional dominion of EBF1 (EBF Transcription Factor 1) and Pax5 (Paired Box 5) factors seal the B cell fate. The expression of RAG1/2 initiates the recombination of the immunoglobulin heavy-chain (IgH) gene and is the first step in the BCR formation and the B cell fate commitment. DSBs are induced by the RAG1/2 complex in specific IgH regions, called recombination-specific regions (RSS). As with every committed cell, the energy demands of these pro-B cells are met through a baseline mitochondrial OxPhos (Figure 3). The hypoxic surrounding microenvironment limits the OxPhos potential of these precursors and ensures that proliferation will not ensue during recombination [27].

### 2.2. From the HSC to the Small Pre-B Cell: Ig Heavy-Chain Recombination

Breaks induced by RAG1/2 are repaired through the cellular DNA repair machinery involving both common as well as lymphoid lineage-specific proteins such as TdT (terminal deoxynucleotidyl transferase) [3]. The end result is a new IgH coding region. The presence of multiple RSS and the inherent flaws of the DNA repair machinery result in an enormous variety of possible recombined IgH loci [28]. Success of the IgH recombination is then tested through binding of the novel IgH chain with a surrogate light chain in a complex that is thereafter called the pre-BCR [28]. If the IgH recombination was indeed successful, signaling through the pre-BCR engages the now-called pre-B cells into a period of rapid proliferation [9,28]. In order to avoid malignant transformation due to genomic instability, this intense proliferation should be coupled with a strict ban of further recombination, and the RAG1/2 complex is silenced [9]. To this end, efficient pre-BCR signaling and existent IL7–IL7R interactions confer a major metabolic swift in the pre-B cell, with massive glycolytic fluxes and moderate increases in OxPhos (Figure 3) [6,9]. The hypoxic microenvironment stabilizes Hypoxia-Inducible Factor 1 (HIF1), the major transcriptional regulator of glycolytic metabolism [29]. Furthermore, IL7 signaling activates the PI3K (phosphoinositide 3-kinases)/Akt (protein kinase B)/mTOR (mammalian target of rapamycin) pathway, ensuring adequate shunting of glycolytic intermediates towards anabolic reactions (Figure 2) [6]. The Akt pathway gives the stabilized HIF1 the green light to perform its metabolic function. Thus, upregulation of glucose transporters and glycolytic enzymes dominate this expanding phase (Figure 3). Warburg glycolysis may be impressively inefficient at providing energy, but it does so with great rapidity [9,11]. Moreover, glucose that is shunted intracellularly is also used to synthesize nucleotides and phospholipids, all required for the upcoming divisions (Figure 2) [9]. A part of the internalized glucose reaches the mitochondria, and engages in OxPhos to add up to the ATP pool and to also provide fatty acids for the expanding cells. Coexistence of both pathways is regulated by the HIF1–Akt/mTor interaction [30]. At this stage, pre-B cells are vitally dependent on glycolysis. Failure to induce the Warburg effect leads to their demise. Filled with ATP and building blocks, the now large pre-B cells expand. Soon, space and nutrients are starting to become limited, ATP reserves drop and pre-BCR signaling becomes dominant over IL7 (Figure 3) [6]. These events both inhibit the PI3K/Akt/mTor pathway and activate the universal energy sensor, AMPK (AMP-activated protein kinase). This kinase negatively regulates the mTor-mediated expanding frenzy while positively affecting mitochondrial biogenesis and TCA substrate (mostly glucose) availability [6,31]. AMPK thus swifts the bioenergetic balance away for aerobic glycolysis towards mitochondrial OxPhos, and this is a pivotal event in the B cell differentiation program as it terminates proliferation at specifically 4–6 cycles [31]. Fnip1, the mediator of AMPK-induced negative regulation of the mTor energy consumption program, was proven essential for the transition from the large to the small pre-B cell [31]. Of note, Fnip1 (Folliculin Interacting Protein 1) was not essential for large pre-B cell proliferation but diminished the ability of the precursors to further differentiate and therefore doomed them to die from energy deprivation [9,29].

Mitochondria are extremely precious for B cells. Maintaining their energy-producing organelles at shape is a constant struggle for the differentiating lineage, and for good reasons. Apart from suppling the cells with energy, as already mentioned, mitochondria are the primary source of oxidative stress (ROS) (Figure 2). During the several genomic modifications, Ig genes lay in an open or untangled chromatin state to be accessible to the proteinic complexes that mediate their recombination [32]. In these open states, they are susceptible to attacking by these nucleophilic species [15]. It is thus crucial for the developing B cells to ensure that if they are to engage in any round of recombination, their mitochondria are not only fit but that they are also able to detect cellular signaling and initiate the apoptotic cascade if anything goes wrong (Figure 2). To this end, a mitochondrial Ca^+2^ sensing channel, EFdh1, is essential for differentiation progression further form the pre-B cell stage. EFdh1 (Swiprosin-2) seems to be downregulated by the pre-BCR signaling, favoring the Warburg metabolism at the large pre-B cell stage, but is essential for further progression to the next developmental stage owing to the subsequent dependence on OxPhos during the Ig light-chain recombination, much like Fnip1 [33]. In a way, metabolic fine-tuning is a prerequisite for differentiation progression. If the small pre-B cell passes all the metabolic checkpoints, the next stage of differentiation initiates and is the one that will provide an immature B cell with a functional IgM-type surface BCR to the periphery (Figure 3).

### 2.3. Immature B Cells and the IgM Assembly

IL7 and pre-BCR signaling cascades intersect at multiple levels, and in general antagonize one another. The strong IL7-IL7R signaling that exists in the IL7-rich pre-B cell niche induces proliferation through cyclin D3 and strong PI3K/Akt activation, though after expression of a functional pre-BCR, it wanes off [6]. The pre-BCR-SYK (spleen tyrosine kinase)-BLNK (B Cell Linker) cascade dominion results in proliferation seize, partially through cyclin D3 inhibition. IL7 downregulation liberates Pax5 and FOXO (forkhead box protein O) transcription factors, thus increasing the accessibility of the Igκ (immunoglobulin light chain) to RAG1/2 complex, that is now re-expressed [6,16,34]. Subsequent downregulation of Cyclin D3 and STAT_5_, and activation of the RAS-ERK pathway, dictate small pre-B cells’ cycle exit. E2A transcription factor recruits the transcriptional co-activators CBP (CREB-binding protein) and p300, and altogether they modify the epigenome of Igκ to make it accessible to RAG1/2 [6,32]. Pre-BCR signaling also positively regulates IRF4 (Interferon Regulatory Factor 4) and IRF8 transcription factors, both of which enable Igκ and Igλ accessibility by epigenetic rearrangements [34].

The second round of recombination that ensues results in a similar expansion blast after successful BCR expression and the release of IgM-expressing immature B cells in the circulation (Figure 3). To ensure that no expansion is carried out during the recombination, metabolic quiescence is induced [9]. The small amounts of ATP required during this stage are supplied by minimal OxPhos activity. The Akt/mTor pathway that was induced by IL7 and mediated the energy burst of the large pre-B cell stage is silenced [6]. Depending exclusively on mitochondrial energy supply renders the cells susceptible to mitochondrial-induced apoptosis, and therefore allows close control of the process [10]. Such a high level of control exists that pre-BCR signaling leads to increased CXCR4 re-expression on the small pre-B cells, that guides them towards new niches with CXCL12 abundance and diminished IL7 expression. In fact, IL7-rich niches lie at great distance from CXCL12 ones, underlining the importance of pathway distinction for maximal control over recombination(s) and proliferation (Figure 1 and Figure 3) [6,23,35].

Successful light-chain recombination, either κ or λ, is tested by the assembly and signaling of the immature BCR, or otherwise the IgM. If the process was successful, strong BCR signaling reactivates the PI3K/Akt axis in a manner similar to IL7 signaling [6]. This leads to a replicative burst similar to that following functional pre-BCR expression. Although at this stage glycolysis is once again upregulated, the expanding immature B cells rely more heavily on OxPhos [9]. Glycolytic inhibition at this stage does not lead to death. If the BCR formed is not functional, PI3K/Akt signaling does not occur, and the cell undergoes further rounds of recombination, first of its Igκ and then of its Igλ genes. Eventually, after a period of expansions, the immature IgM^+^ B cells are liberated from BM to the periphery to continue their training program (Figure 3).

### 2.4. Lymph Nodes: Overview of the B Cell Expansion Maturation and Fate Choice Decision

As soon as they settle in the secondary lymphoid organs (for simplicity in this review, the lymph nodes (LNs)), immature B cells have to face a second round of extensive genomic modifications followed by their selection and expansion. This process will allow for the formation of both antibody-secreting plasma cells (PCs) and memory B cells (MBCs).

**Figure 4:**
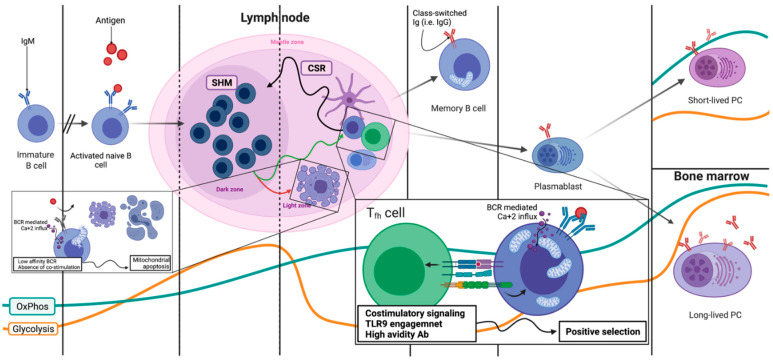
B cell differentiation completion in secondary lymphoid organs. The GC reaction is shown. B cells undergo multiple rounds of SHM in the dark zone (DZ) and then positive selection in the light zone (LZ) until the avidity of their surface BCR is adequate to guarantee them class switching and either the memory B cell or plasma cell fate. Positive (or negative) selection occurs through a mitochondrial-based mechanism that involves Ca^+2^ influx due to BCR signaling. If the BCR/Ab does not bind with high avidity to the stimulating Ag, or if there are no costimulatory signals (either T cell-derived or TLR9), mitochondria internalize the cytoplasmic Ca^+2^ and the apoptotic cascade described in Figure 1 is initiated. Co-stimulations or high avidity initiate a rescuing cascade, that involves NF-κΒ activation and antiapoptotic protein expression, that rescues the B cell and allows further entry into the DZ for subsequent modifications. Again, energy modulation and bioenergetic swifts regulate proliferation and genomic editing and are depicted in the lower part of the figure (orange—glycolysis, blue—OxPhos). SHM: somatic hypermutation, CSR: class switch recombination, Tfh: T follicular helper cell, TLR9: Toll-like receptor 9. Created with BioRender.com.

After clonal selection in the BM, the immature B cells that are tested start an as of today elusive process of further maturation, characterized by low glycolysis and moderate OxPhos (Figure 3 and Figure 4) [9]. These now naïve mature B cells can remain quiescent in the mantle zone of a primary, not activated, lymphoid follicle. If the naïve mature B cell is stimulated by an Ag, two very different fates await: either immediate expansion and differentiation towards an IgM-secreting B cell or germinal center (GC) entry, GC reaction and maturation [9,36]. During the GC reaction, the activated B cell undergoes several rounds of expansion and further BCR editing that culminate in class switch recombination (CSR) [28]. The genomic Ig sequences undergo further modifications, in sum called somatic hypermutations (SHM), to refine the BCR and achieve maximal avidity for the Ag that activated the B cell [28]. All of these events which include DSB are performed inside secondary lymphoid follicles (Figure 4). Apart from involving different parts of the Ig genes, CSR and SHM pose another major difference. The DSB that are induced in each process are repaired through two very distinct mechanisms. CSR is achieved by non-homologous end-joining (NHEJ), which is implemented only during the G1 phase of the cell cycle. On the contrary, the SHM deploys the homologous recombination (HMR) during the S/G2 phase. Given that both CSR- and SHM-related DSBs are induced by the same enzyme, activation-induced cytidine deaminase (AID), spatial distinction of the B cells in G1 from those in S/G2 seems a very efficient way to control what happens and when it occurs [37]. Of course, epigenetic alterations and differential transcription factor expression (EP300 vs. CREBBP for instance) ensure that at each location, only the desired part of the Ig gene will be accessible for AID to induce the DSBs [38].

### 2.5. Lymph Nodes: Light Zone, Dark Zone, BCR Refinement and Final Differentiation

Secondary lymphoid follicles are divided into two distinct areas. One, named the light zone (LZ), is the area where activated naïve B cells antagonize each other for T cell help in order to survive and further differentiate [7]. While in LZ, the B cells, now called centrocytes, do not proliferate. Signaling through their BCRs leads to Ca^+2^ being released for their endoplasmic reticulum (ER) [39]. Depending on the strength of BCR-Ag binding and/or other Ag properties, the strength of this Ca^+2^ wave differs [40]. Meanwhile, again through BCR signaling, an increase in the centrocyte’s mitochondrial mass is observed [9,41]. However, the LZ microenvironment is largely hypoxic and nutrient-deprived. That means that the centrocytes are prepared to boost their metabolism but are deprived of both oxygen for OxPhos and nutrients to perform glycolysis. Lack of oxygen and Ca^+2^ release pose a substantial stress for the centrocyte’s increased mitochondrial mass. If these cells do not receive co-stimulatory signals from surrounding T cells (CD40/CD40L interaction or TLR9 co-engagement), they die by mitochondrial apoptosis (Figure 4) [10,36]. This cruel selection scheme allows only for the most competent BCR clones to survive and also protects against autoreactivity from unspecific clones. There is success in receiving the second signal, and the centrocytes are rescued by the expression of c-Myc, the major pro-survival B cell transcription factor, and direct NF-kB (nuclear factor kappa bet) stimulation [39]. The expression of c-Myc along with further co-stimulation eventually leads to further metabolic reprogramming. Upregulation of glycolytic transporters, as well as increased aerobic glycolysis and increased mitochondrial OxPhos, mark the centrocytes’ migration towards the GC dark zone (DZ) for further proliferation (Figure 4) [9,10,41]. Oxygen and nutrient availability along with chemokines may dictate this migratory wave. Activation of mTor, this time from c-Myc, enables anabolism for the upcoming expansion [42]. When in DZ, centroblasts utilize glucose for anabolic pathways through a c-Myc-induced Warburg effect, while mitochondria utilize alternative carbon sources, such as glutamine and fatty acids, to perform OxPhos (Figure 2) [41,43]. Mitochondrial reorganization and fatty acid oxidation characterizes GC B cells and is a metabolic signature that they retain even in their terminally differentiated state, PC or MBC.

If the Ca^+2^ wave is of substantial height, for instance through binding with T-cell-independent antigens, polysaccharides and TLR9 ligands’ CpG motifs, the centrocytes do not need any further stimulation [10,36]. Through as yet poorly understood pathways, the height of the Ca^+2^ is enough to activate pro-survival pathways (Akt and NF-kB, as well as BCL2) and thus rescue the cells from demise [44]. Strong signals bypass the BCR specificity requirement and lead to the IgM-secreting B blasts that are part of the innate immunity.

In the DZ, centroblasts expand and SHM takes place. A poorly understood and complex regulatory network of positive and negative feedbacks ensures that this expansion is timely regulated. After somewhat 8 cycles of proliferation, centroblasts migrate again to the LZ to test their newly mutated BCRs; again, those positively selected will reenter the DZ [42]. Within each cycle of LZ, DZ transitioning the same metabolic swifts occur. Each cycle gives raise to both short-lived, antibody secreting, class-switched PCs (SLPC) in the LZ, and MBCs. Once again, metabolic profiles differ impressively to match the terminal fate of the B cell. SLPC depend heavily on OxPhos for their energy requirements (Figure 4). They present a vastly upregulated autophagic drive and ER response stress machinery to compensate for the enormous amounts of unfolded proteins, namely Ab that accumulate in their ER during their short lifetime [8,45]. MBCs on the other hand are practically quiescent [45]. They perform minimal amounts of both glycolysis and OxPhos and remain dormant until Ag binding engages them in a second round of GC maturation. GC B cells rely on mitochondria, and not only for ATP production. Several studies have shown that GC B cells actively oxidize fatty acids in the TCA, while glucose is shunted away from mitochondria towards the aerobic pathway, with the benefits that have already been discussed. To this end, IL4, a major pro-survival cytokine from the GC microenvironment, has been shown to induce fatty acid oxidation (FAO) in the TCA and to modify the enzymes involved in the cycle to accumulate the metabolite a-ketoglutarate (aKG) (Figure 1 and Figure 2) [27,46]. aKG that is produced directly modifies the epigenome of a major GC pro-survival cytokine Bcl6 to allow for its expression by acting as a co-factor for the UTX H3K27-demethylase. Histone 3k27 demethylation by UTX then allows for STAT_6_ to bind and activate the Bcl6 locus, and this pathway is indispensable of GC B cell fate [47]. Bcl6 is a transcription factor essential for GC B cell survival [48,49]. Among its many functions, it increases DNA repair capabilities and renders cells more resistant to death due to DNA damage. Both functions are crucial for healthy as well as malignant B cell survival [48]. Bcl6 is found hypermutated and hyperactivated very often in B cell lymphomas [48]. Heme, an iron-containing porphyrin that is used as the oxygen carrier from ETC and is actively synthesized almost exclusively inside the mitochondrial matrix, has also been shown to play an important role in B cell differentiation [46]. It actively suppresses Bach2, a transcription factor that promotes Blimp1 expression and thus PC differentiation [50]. By doing so, heme allows for both SMH and CSR to occur before further differentiation is allowed. Heme directly regulates both DNA binding and protein stability of Bach2 (BTB Domain and CNC Homolog 2) by inducing its export to the cytoplasm and subsequent polyubiquitination and degradation [46,50]. Thus, the metabolic reprogramming that is induced via BCR activation and that leads to mitochondrial biogenesis and heme accumulation dictates B cell fate, regulating metabolic reprogramming.

After multiple rounds of GC DZ-LZ transitioning through a very specific transcriptional programming involving Blimp1, and only after having achieved maximal Ag specificity for their class-switched BCR, the elite of PC arise from the GC [51]. These are the long-lived PC (LLPC). In contrast to their SL brothers, LLPCs secrete much higher amounts of Ab and can live for decades inside the BM, where they migrate without dividing [52,53].

## 3. Metabolism of the Most Common B Cell Malignancies

The field of metabolomics in B cell malignancies is only now starting to dawn. Very little data exist on the metabolic profile of B cell-derived tumors. Important steps are being taken though, and the importance of metabolic alteration both as a means of prognosis as well as an Achilles tendon for novel therapies is increasingly becoming evident [21,54]. In the following section, we will summarize what is known for the most common B cell-derived malignancies, relating their profiles to the normal counterparts of the malignant cell.

### 3.1. Classical Hodgkin’s Lymphoma (cHL)

The lymphomatous cell of cHL, the Reed-Sternberg cell (RS), is considered to be an apoptotic B cell that withstood death signaling from its surrounding microenvironment and survived [55]. This theory also explains the observed reactivity of the microenvironment surrounding the RS cells. From the bioenergetic aspect, RS cells have shown increased expression of the lactate transporter MCT1 (monocarboxylate transporter 1) and high mitochondrial mass. Moreover, their surrounding microenvironment, especially tumor-associated macrophages and stromal cells, engages in paradoxical glycolysis and expresses the lactate exporter MCT4 [56]. These relationships that develop between the RS and their microenvironment are increasingly being identified in several malignancies and are summarized under the term reverse Warburg effect [57]. Glycolysis performed on the surrounding stroma supplies the RS cells with substrates, for instance lactate imported through MCT1, to perform OxPhos [58]. This highly beneficial relationship is so balanced that oxygen consumption from the OxPhos of the RS cell drops the oxygen tension in the microenvironment, engaging the stromal cells in the Warburg metabolism (Figure 2). Of course, anabolic substrates produced in this process by the stroma could be exported and utilized by the malignant cells, which now act only for energy production through OxPhos [11]. Studies are scarce and further research is needed to shed light on these complex metabolic relationships, but it seems that as with almost all malignancies, cHL is as much a B cell as well as a stromal disease, something that should be taken into account when designing therapeutic approaches.

### 3.2. Multiple Myeloma (MM)

Long-lived PC (LLPC) have long been considered the cell of origin of both MM and MGUS (monoclonal gammopathy of undetermined significance). Much like LLPCs, MM PCs rely substantially on the BM microenvironment to provide them with survival signals and the much-needed nutrients [1]. In comparison with their more futile and transient short-lived counterparts, SLPC, both MM and LLPC acquire a unique bioenergetic profile that allows them to compensate for the profound stress that Ab secretion bears [52,53]. A mixture of both OxPhos and glycolysis characterize their metabolome with glycolysis, suppling not only reducing power and nucleotides, but most vitally, the much-needed phospholipids that will be used for ER synthesis [52]. In the process of Ab secretion, vast amounts of ER are consumed through autophagy by means of the ER stress response pathway. The ability of LLPCs and MM cells to unlock this complex metabolic program differentiates them from the SLPCs [53]. Anaplerosis of the TCA cycle with glutamine and the downstream production of oncometabolites such as hydroxyglutarate, as well as release of immunomodulating ones such as adenosine (ADO), have also been documented in MM [59]. Of course, as the disease progresses from the MGUS to MM, increased independence from the BM microenvironment and increased modulation of the niche is observed. To this end, evidence exists that stromal BM cells support the growth of MM cells through mitochondrial transfer [60,61]. Reverse Warburg and metabolic coupling of the MM cell with the microenvironment may modulate this organelle exchange (Figure 1).

### 3.3. Diffuse Large B Cell Lymphoma (DLBCL)

DLBCL is an umbrella term describing a wide variety of aggressive B cell neoplasms comprised of large basophilic, rapidly proliferating B cells. The pathologic finding of these large cells along with a diffuse disruption of the underlining stroma gave this category its distinct name [62]. The first approach to characterize them yielded three distinct groups based on their gene expression profile (GEP) in relation to their cell of origin (COO approach). Germinal center DLBCL (GC DLBCL), which exhibit a transcriptional profile compatible with ongoing SHM and GC B cell origin, were the first and more benign group. The second and much more aggressive group, the activate B cell (ABC DLBCL), showed a phenotype of a post-GC B cell trapped just before PC differentiation at the plasmablast stage [63]. This also explains the downregulation of MHCII molecules found in this subtype, as PC differentiation physiologically coincides with downregulation of the antigen-presenting features of the B cell. The third group comprising approximately 30% of DLBCL tumors was not compatible with any specific B cell phenotype and was thus classified as unclassified DLBCL [63].

With the increasing evidence that microenvironmental influence is a major determinant of tumor prognosis and therapy response, a second GEP approach, the consensus cluster classification (CCC), managed to re-categorize DLBCL tumors based on their stromal elements and yielded again three distinct subgroups with as yet no difference in prognosis. This approach, however, may help to identify new therapeutic targets, as it examines a clearly functional aspect of the tumor and is therefore worth mentioning [64]. The first cluster identified was termed “OxPhos DLBCL” due to the dominant presence of genes related to mitochondria. Upregulation of cytochrome c/cytochrome c oxidase complex (COX), NADH dehydrogenase, mitochondrial ribosomal subunits and members of the Bcl2 family clearly show that in this group, mitochondrial health and function is of primary importance and can therefore be potentially targeted. The second subgroup, termed “BCR/Proliferation DLBCL”, was defined by marked upregulation of cell cycle regulators and dominion of the BCR signaling cascade. The third group differed from the other two in that it showed a signature defined by the associated host response and not by the tumor itself, and was thus termed “Host response DLBCL” [62]. Bioenergetically, DLBCL remains as complicated and as diverse as these classification attempts underline. Dependence on OxPhos has been shown and was to be expected, given the GC B cell origin [65]. However, the malignant cells have also been suggested to engage their microenvironment to the reverse Warburg effect, as much as the cHL RS do [66,67]. Moreover, increased resistance to mitochondrial apoptosis has also been documented. To this end, a decoupled nuclear and mitochondrial OxPhos regulation under hypoxia seems to drive this apoptotic resistance. Much is to be discovered for DLBCL [67,68]. Intra- and inter-tumor heterogeneity make establishing metabolic features of these tumors extremely difficult [64].

### 3.4. Chronic Lymphocytic Leukemia (CLL)/Small Lymphocytic Lymphoma (SLL)

The CLL/SLL cell of origin has been and still remains a conundrum. Depending on the presence or not of IgH hypermutation, the CLL/SLL cases have been traditionally divided into a mutated (M-CLL) and an unmutated (U-CLL) subgroup, the latter with much worse prognosis [69]. Although for M-CLL the cell of origin has been considered a GC derived MBC, for the unmutated group, a T-cell-independent marginal zone B cell has been blamed [69]. In any case, early mutation and/or non-coding RNA abnormalities as early as the HSC stage have been considered to predispose the subsequent clones to further events that lead to leukemic/lymphomatous transformation. Metabolically speaking, data are scarce. For the better-studied M-CLL group, a marked predisposition of the circulating cells to FAO and mitochondrial OxPhos confirm that mutated CLL cells have passed through the GC reaction [70]. On the other hand, the much more aggressive U-CLL cells show increased glycolytic reserve along with a transcriptional signature compatible with increased reliance on BCR signaling [70]. An existing theory states that as the CLL cells transition from circulation to LN (or other organ) homing, they need to adapt their metabolism to the microenvironmental conditions [71]. Moreover, as circulating CLL cells have been considered not to divide, it is possible that the higher glycolytic reserve of U-CLL reflects their higher dividing potential upon reaching their niche, a fact that can also account for the inverse relationship between glycolytic potential and prognosis. The increased metabolic flexibility and glycolytic potential can also account for acquired resistance to mitochondrial-targeting therapies, such as the anti-Bcl2 agent, venetoclax, a mainstay of anti-CLL treatment nowadays [72].

### 3.5. Mantle Cell Lymphoma (MCL)

MCL is a rare mature B cell neoplasm characterized by the t(11;14) translocation that juxtaposes cyclin D1 on chromosome 11 under the transcriptional influence of Ig on chromosome 14 [73]. Overexpression of cyclin D1 that follows along confers a survival and divisionary advantage to the mutated clones. The increased dividing drive leads mutations to further accumulate until the full-blown MCL develops [73]. MCL depend on downstream BCR signaling for survival. Bruton’s tyrosine kinase (BTK), a mediator of the BCR signaling pathway, has been repeatedly and effectively targeted for the MCL treatment. Dependency on the BCR somewhat reflects on the metabolic profile of these tumors, as similar to their normal counterparts, they show a predilection for glycolysis and concomitant mitochondrial oxidation of the in-fluxed glucose [22,74]. Either due to BTK mutations or via overactivation of pro-survival pathways, such as NF-kB and/or the PI3K/Akt/mTor, MCL can become resistant to BTK inhibitors [74]. These tumors show a predilection for OxPhos, minimal glycolysis and mitochondrial anaplerosis with glutamine [74]. Although targeting of the mTor pathway would seem promising for such tumors, clinical results were disappointing [75]. It is possible that as with other malignancies, metabolic changes are driven by a complex network of interacting and intersecting signals that offer extreme plasticity to the newly formed balance.

### 3.6. Follicular Lymphoma (FL)

FL is an indolent B cell-driven lymphoma that is considered generally incurable. A minor subset of them transform into DLBCL with much graver prognosis than a de novo occurring DLBCL. Bcl2, a mitochondrially related pro-survival protein, is often found overexpressed in FL [19]. In these cases, Bcl2 juxtaposition under the Ig promoter, the t(14;18) transposition, is the prototypical genetic event that leads to its overexpression. The mutated cells with the Bcl2-driven survival benefit undergo GC reaction and dominate among the normal clones. Bcl2^+^ FL is profoundly dependent on the GC microenvironment for survival [19]. The heterogeneity observed in FL Ig genes denotes ongoing SMH. Eventually, clonal selection occurs for the most rapidly proliferating and less immunogenic clones [76]. These clones that have acquired further genetic aberrations are gradually dominating. Eventually, major events such as CDKN2A/2B inactivation, c-Myc mutation or TP53/Bcl6 mutation render these clones independent of Ag stimulation and microenvironmental support and lead to DLBCL transformation [19]. Bioenergetically, indolent FL cells might show a profile similar to normal GC B cells with fatty acid oxidation and dominant OxPhos [22]. Upon transformation however, a more glycolytic profile ensues [77]. Upregulation of glycolytic biomarkers has been suggested as a marker for DLBCL transformation, but in-depth studies are still lacking.

## 4. Conclusions

Bioenergetic fluctuations are indispensable for every cell’s fate. In the B cell physiology however, metabolic alterations regulate and are regulated in a constant negative/positive feedback loop, the cellular differentiation program. This happens to ensure that genomic modifications do not coincide with pro-survival signaling and proliferation. Despite the tightly regulated B cell differentiation scheme, malignant transformation does occur. The field of metabolomics in B lymphoid malignancies is still in its infancy. Scattered data exist that roughly characterize lymphomatous B cells and have to date failed to become clinically relevant. Progress is made as it is becoming increasingly evident that metabolic drifting apart from the physiologic analogue often translates to progression to a more aggressive disease. Apart from this clearly prognostic aspect, knowledge of the metabolic profile of the malignant cell, both isolated as well as within its microenvironment, may alter our therapeutic approach into targeting such vital bioenergetic pathways. Moreover, as metabolism possesses extreme plasticity and can directly affect the genome/epigenome, bioenergetic swifts may become drivers of further tumor progression, diversity, immune evasion and graver clinical course. Many steps are still to be taken in order for us first to understand and then to be able to manipulate metabolic pathways in the anti-lymphoma armamentarium.
